# Optimising the detectability of H5N1 and H5N6 highly pathogenic avian influenza viruses in Vietnamese live-bird markets

**DOI:** 10.1038/s41598-018-37616-1

**Published:** 2019-01-31

**Authors:** Timothée Vergne, Anne Meyer, Pham Thanh Long, Doaa A. Elkholly, Ken Inui, Pawin Padungtod, Scott H. Newman, Guillaume Fournié, Dirk U. Pfeiffer

**Affiliations:** 1 VEEPH Group, Royal Veterinary College, Hatfield, United Kingdom; 2 MIVEGEC Group, Institut de Recherche pour le Développement, Montpellier, France; 3 UMR IHAP, Université de Toulouse, INRA, ENVT, Toulouse, France; 4 Department of Animal Health, Ministry of Agriculture and Rural Development, Hanoi, Vietnam; 5 Food and Agriculture Organization of the United Nations, Hanoi, Vietnam; 6 Centre for Applied One Health Research and Policy Advice, City University of Hong-Kong, Hong-Kong SAR, PR China

## Abstract

Live bird markets (LBMs) are major targets for avian influenza virus (AIV) surveillance programmes. While sampling the LBM environment has become a widely used alternative to the labour-intensive sampling of live poultry, the design of surveillance programmes and the interpretation of their results are compromised by the lack of knowledge about the effectiveness of these sampling strategies. We used latent class models and a unique empirical dataset collated in Vietnamese LBMs to estimate the sensitivity and specificity of five different sample types for detecting AIVs subtypes H5N1 and H5N6: oropharyngeal duck samples, solid and liquid wastes, poultry drinking water and faeces. Results suggest that the sensitivity of environmental samples for detecting H5N1 viruses is equivalent to that of oropharyngeal duck samples; however, taking oropharyngeal duck samples was estimated to be more effective in detecting H5N6 viruses than taking any of the four environmental samples. This study also stressed that the specificity of the current surveillance strategy in LBMs was not optimal leading to some false positive LBMs. Using simulations, we identified 42 sampling strategies more parsimonious than the current strategy and expected to be highly sensitive for both viruses at the LBM level. All of these strategies involved the collection of both environmental and oropharyngeal duck samples.

## Introduction

Highly pathogenic avian influenza (HPAI) viruses continue to threaten local economies, famers’ livelihood and food security in countries where they are considered endemic such as China, Vietnam, Egypt or Bangladesh^1–4^. They are also a threat to HPAI-free countries where they may be introduced and cause epidemics, as recently experienced in Europe^5^. Due to their potential to reassort with human influenza viruses, some avian influenza viral strains are also a serious threat to public health^6^. For these reasons, monitoring the circulation of avian influenza viruses (AIVs) is of paramount importance.

It is now widely acknowledged that trade of live birds plays a major role in the spread of AIVs. Live-bird markets (LBMs) have regularly been found contaminated in endemic contexts^7–12^ and it has often been stressed that AIVs are more frequently detected in LBMs than in farms^13^. Therefore, they represent a prime location where implementing surveillance activities is extremely convenient. In addition, LBMs pose a real threat to public health as they may promote both the amplification of the virus and close contacts between poultry and humans^7,14,15^. Consequently, LBMs are locations where implementing appropriate targeted interventions can be highly effective for preventing disease spread along the trading network and mitigating the public health risk posed by AIVs^16,17^.

Surveillance for AIVs in LBMs is often conducted by collecting oropharyngeal or cloacal swabs directly from the live poultry sold in these markets^8,10,18–20^. However, sampling live birds is generally poorly accepted by farmers and traders^21,22^, as it may create fear among customers about the health status of sampled birds and therefore decrease the economic value of these animals. Environmental samples, involving the collection of materials such as faeces or dust, allow the detection of various AIV subtypes in contaminated LBM and are therefore often considered as a useful alternative to oropharyngeal or cloacal bird samples^10,11,23,24^.

In a context of limited budgets allocated to infectious disease surveillance, the scientific community regularly stresses the need to optimise the monitoring of AIVs in LBMs by identifying the materials most likely to test positive in contaminated LBMs^10,21^ and designing more sensitive diagnostic tools^25^. Knowing the performance of different sample types for detecting AIVs is crucially important for both designing optimised surveillance strategies in LBMs and interpreting surveillance outcomes while accounting for imperfect detection processes. Despite the growing popularity of environmental samples for detecting AIVs, there is no quantitative evidence of the effectiveness of different environmental sampling strategies in comparison to live bird sampling. As an illustration, Chen *et al*.^12^ showed that the overall proportion of H7N9-positive samples collected over 11 months in 50 surveillance sites (including 42 LBM and 8 farms) from Guangzhou, China, was significantly higher amongst environmental samples than live poultry samples. However, the methodological framework used to generate these results could not account for the presence of false positives, false negatives nor for potential dependence between sample types. Therefore, authors could not infer quantitative estimates of the effectiveness of each strategy in identifying contaminated LBMs.

In this paper, we present a robust statistical framework relying on latent class models for analysing the outcomes of complex surveillance programmes that use several sample types for detecting AIVs in LBMs. We illustrate the approach using empirical data generated by the Vietnamese surveillance programme, for which oropharyngeal duck samples and four different types of environmental samples were collected to monitor the circulation of HPAI H5N1 and H5N6 subtypes in LBMs over a 14-month period. The objectives of the study were 1) to estimate quantitatively the effectiveness of these different sample types for detecting these two HPAI subtypes and 2) to identify the optimal combinations of sample types that maximise the detectability of both H5N1 and H5N6 subtypes in Vietnamese LBM while minimising the number of collected samples.

## Material and Method

### Ethics statement

The duck sampling protocol was approved by the Department of Animal Health of the Vietnamese Ministry of Agriculture and Rural Development (MARD) and carried out in accordance with licences from the MARD.

### Live-bird market survey

**Figure 1:**
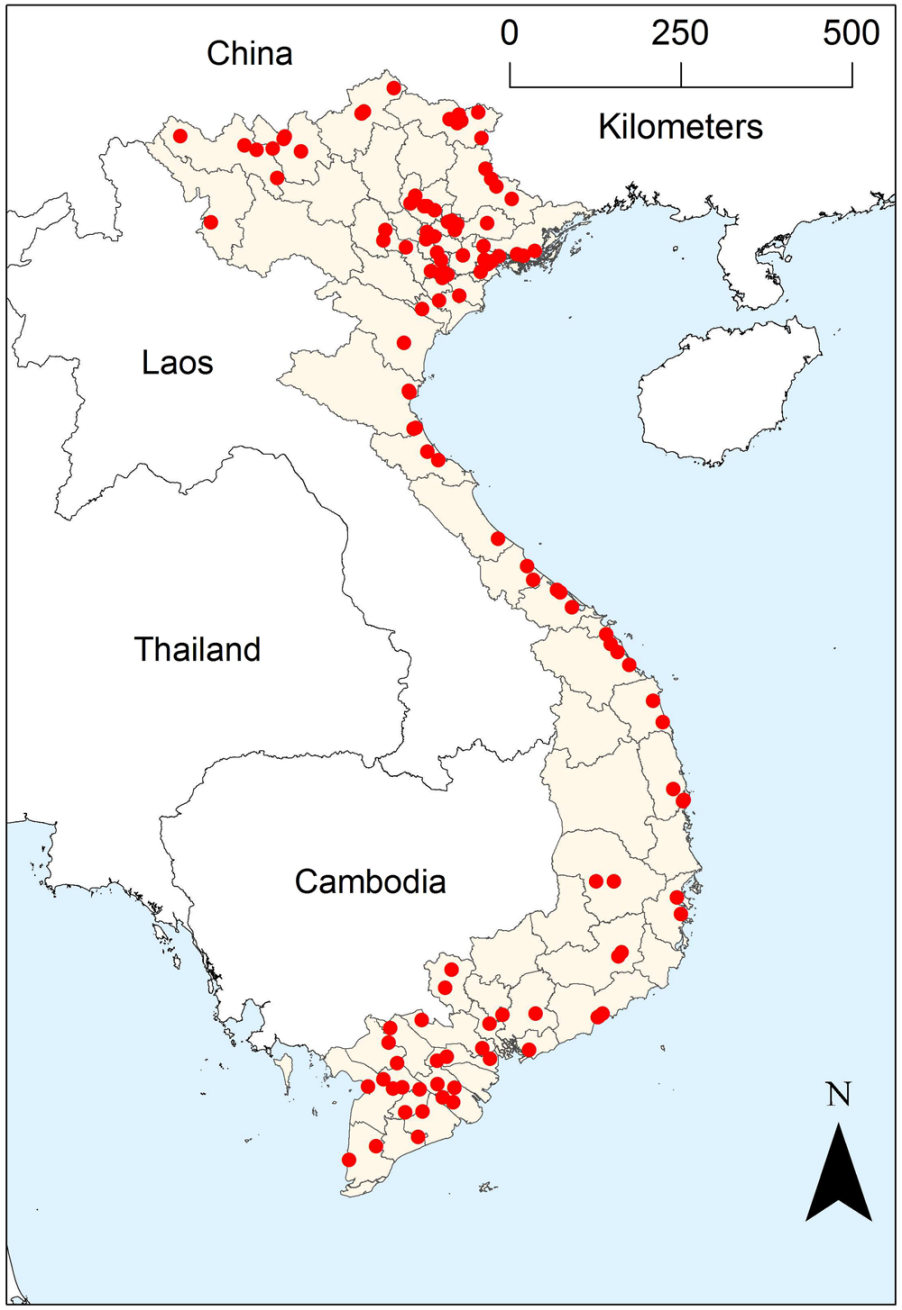
Spatial distribution of the live bird markets involved in the avian influenza surveillance activities in Vietnam between November 2014 and December 2015. Red dots represent the selected LBMs, Vietnam is shaded in light brown while the sea is highlighted in light blue. This figure has been produced using ArcMap version 10.1^44^.

As part of the avian influenza virus national surveillance plan, the Department of Animal Health of the Ministry of Agriculture in Vietnam implemented a nation-wide LBM survey between November 2014 and December 2015 and visited 140 markets distributed all across the country (Fig. 1). To be included in the survey, LBMs were required to have at least 6 duck vendors.

In each visited LBM, five different sample types were collected as follows: 30 oropharyngeal duck samples, 10 solid waste samples, 10 liquid waste samples, 5 drinking water samples and 5 fresh faeces samples (Table 1). The oropharyngeal duck samples were collected amongst 6 randomly selected duck vendors, with five ducks being sampled per vendor. Samples were pooled by five, respective to their type, directly in the market. Consequently, there were 12 pooled samples per visited LBM. Because a pooled sample is the unit of the analysis, in the subsequent sections of the manuscript, we refer to them simply as the “samples”. All samples were screened for the influenza A matrix gene using rRT-PCR at the closest regional veterinary laboratory; all positive samples were then tested for the H5 gene; finally, all H5-positive samples were tested for N1 and N6 genes. LBM visits for which the number of collected samples per sample type was less than expected (Table 1) were discarded from the analyses.

**Table 1:** Description of the five sampling protocols implemented in each Vietnamese live-bird market involved in the surveillance activities between November 2014 and December 2015.

ID of the sampling protocol	Sampling type	Description of the sample type	Number of individual samples	Number of pooled-by-five samples
1	Duck	Oropharyngeal swab	30	6
2	Solid waste	Swab of dust taken on the floor of poultry cages	10	2
3	Liquid waste	Swab of waste water taken in drain channels	10	2
4	Drinking water	Swab of drinking water in water troughs	5	1
5	Faeces	Swab of fresh faeces taken in duck resting areas	5	1

The survey was primarily designed as a longitudinal study, so most LBMs were visited at least twice during the survey period. Consequently, to avoid dependence between observations, successive visits of the same market were included in the analysis only if they were implemented at least 10 days apart from each other. Otherwise, only the first of the two visits was included in the analysis. This assumption was considered to be acceptable since (i) the poultry population turnover in LBMs is sufficient for assuming that the viral circulation could not be maintained amongst the birds during more than a few days, (ii) avian influenza viruses are unlikely to persist for that amount of time in such environment^26,27^. The influence of this assumption on the study results was assessed in a sensitivity analysis by using 15 and 20 days instead of 10.

For a given virus subtype (H5N1 or H5N6) and a given visit, a LBM was considered to have been detected as contaminated by the duck sampling protocol if at least one of the six oropharyngeal duck samples was tested positive for that subtype. Similarly, a LBM was considered to have been detected as contaminated by the solid waste sampling protocol if at least one of the two solid waste samples was tested positive. Consequently, a LBM was considered to have been detected as contaminated by both the duck sampling protocol and the solid waste sampling protocol if at least one of the six duck samples and at least one of the two solid waste samples were tested positive. All cross-detection combinations between the five sample types were defined similarly.

### Latent class analysis

The latent class modelling approach that was used to model the outcome of the LBM survey has been extensively applied to analyse cross-detection of individuals whose true epidemiological status (infected or non-infected) is assessed using at least two imperfect diagnostic tests of unknown sensitivity and specificity^28–31^. In this study, we adapted this classic modelling approach to analyse cross-detection of LBMs whose true epidemiological status (presence or absence of H5N1 or H5N6 virus) was assessed using five different imperfect observation processes (based on five different sample types) of unknown sensitivity (defined as the probability of detecting the virus given it is present in a LBM) and specificity (defined as the probability of not detecting the virus given it is absent from a LBM).

Let D be the true (and unobserved) epidemiological status of a LBM at the time of the visit such that D = 1 if the LBM is contaminated, *i*.*e*. if at least one bird is infected by the AIV strain of interest or if the virus is present in the LBM environment and D = 0 otherwise. The prevalence π is defined as the probability that a LBM is truly contaminated at the time of the visit, which corresponds to the proportion of contaminated LBMs in the population of LBMs under study at the time of the visit, *i*.*e*. π = P(D = 1). Let T_i_ be the test result for the *i*th sampling protocol (i = 1, 2,… 5), such that T_i_ = 1 denotes that at least one sample of type *i* is tested positive, and T_i_ = 0 denotes that all samples of type *i* are tested negative. The sensitivity of the sampling protocol *i* (Se_i_) is the conditional probability that at least one sample taken as part of the *i*th sampling protocol in a contaminated market is tested positive, *i.e.* Se_i_ = P(Ti = 1 | D = 1). Similarly, the specificity of the *i*th sampling protocol (Sp_i_) is the conditional probability that all samples taken as part of this sampling protocol in a non-contaminated market are tested negative, *i.e.* Sp_i_ = P(Ti = 0 | D = 0). For a given sampling protocol *i*, Se_i_ and Sp_i_ can be expressed as follows:

$${{\rm{Se}}}_{{\rm{i}}}=1-{(1-{{\rm{Se}}}_{{\rm{i}}\_{\rm{sample}}})}^{{\rm{n}}}$$

$${{\rm{Sp}}}_{{\rm{i}}}={{{\rm{Sp}}}_{{\rm{i}}\_{\rm{sample}}}}^{{\rm{n}}}$$

with n being the number of samples taken as part of the *i*th sampling protocol (see Table 1) and Se_i_sample_ (respectively Sp_i_sample_) being the sensitivity (resp. specificity) of the *i*th sampling protocol at the sample level, *i*.*e*. the conditional probability that a sample taken as part of the *i*th sampling protocol in a contaminated (resp. non-contaminated) LBM is tested positive (resp. negative). This formulation allowed us to account for the varying number of samples between sampling protocols. It is worth noting that (i) Se_i_sample_ can be seen as a combination of the probability that a sample collected as part of the *i*^*th*^ sampling protocol in a contaminated LBM is contaminated and of the intrinsic sensitivity and specificity of the diagnostic test used on that sample type, and (ii) that Sp_i_sample_ is equivalent to the intrinsic specificity of the diagnostic test used.

The observed frequency of the 2^5^ = 32 different combinations of test results (Table 2) was assumed to be distributed according to a multinomial distribution of parameters N = 230 visited markets and 32 probabilities expressed as a combination of π, Se_i_sample_ and Sp_i_sample_. Conditional dependence between specific sampling protocols in contaminated and non-contaminated markets was modelled by adding covP and covN, respectively, which are parameters that correspond to the covariance between the two assumed-dependent sampling protocols, as described in Dendulkuri and Joseph (2001)^32^. As an illustration, the model accounting for an interaction between the sampling protocols 1 and 2 is given as a Supplementary Methods.

**Table 2:** Distribution of the 32 cross-classified results of the five sampling protocols for H5N1 and H5N6 subtypes.

Sampling protocol					H5N1		H5N6	
Ducks	Solid waste	Liquid waste	Drinking water	Faeces	Observed	Predicted*	Observed	Predicted**
0	0	0	0	0	204	189–209	187	172–195
1	0	0	0	0	10	5–18	20	13–29
0	1	0	0	0	2	0–6	1	0–5
0	0	1	0	0	5	2–11	3	1–8
0	0	0	1	0	0	0–4	0	0–2
0	0	0	0	1	1	0–5	1	1–4
1	1	0	0	0	0	0–3	5	1–7
1	0	1	0	0	2	0–4	2	1–5
1	0	0	1	0	1	0–2	1	0–1
1	0	0	0	1	1	0–2	0	0–1
0	1	1	0	0	0	0–1	0	0–1
0	1	0	1	0	1	0–1	0	0–0
0	1	0	0	1	0	0–1	0	0–0
0	0	1	1	0	1	0–1	1	0–3
0	0	1	0	1	0	0–1	0	0–0
0	0	0	1	1	0	0–2	0	0–0
1	1	1	0	0	1	0–1	5	2–9
1	1	0	1	0	0	0–1	0	0–3
1	1	0	0	1	1	0–1	0	0–2
1	0	1	1	0	0	0–1	1	0–2
1	0	1	0	1	0	0–1	0	0–1
1	0	0	1	1	0	0–1	0	0–0
0	1	1	1	0	0	0–0	0	0–0
0	1	1	0	1	0	0–0	0	0–0
0	1	0	1	1	0	0–0	0	0–0
0	0	1	1	1	0	0–0	0	0–0
1	1	1	1	0	0	0–1	1	0–4
1	1	1	0	1	0	0–1	1	0–2
1	1	0	1	1	0	0–1	0	0–1
1	0	1	1	1	0	0–1	0	0
0	1	1	1	1	0	0–0	0	0
1	1	1	1	1	0	0–0	1	0–1

The predicted values correspond to the 95% credible interval of the posterior distributions rounded to the nearest unit, as predicted by the best-fit Bayesian latent-class models.

^*^The best-fit model for H5N1 did not include any interaction between any of the sampling protocols.

^**^The best fit model for H5N6 included a positive interaction between the liquid waste and the drinking water sampling protocols in non-contaminated live bird markets.

The analyses were performed independently for H5N1 and H5N6 in a Bayesian framework using the WinBUGS software^33^ embedded in the R software^34^ by the R2WinBUGS library^35^. We assumed Uniform(0, 1) as prior distributions for π and the Se_i_sample_ parameters. Since the rRT-PCR testing was considered to be highly specific (*i.e.* non-contaminated samples are very likely to test negative), Sp_i_sample_ parameters were assigned a beta prior distribution defined such that its 5^th^ percentile was equal to 80%, and its median to 98%. The covariance terms (covP and covN) were given uniform distributions whose parameters were specified as in Dendulkuri and Joseph (2001)^32^. As an illustration, the covariance terms between protocols 1 and 2 in contaminated markets and non-conaminated markets (covP_12_ and covN_12_, respectively) were given uniform distributions defined by:

$$\begin{array}{c}{{\rm{covP}}}_{12} \sim Uniform\,(max(-(1-Se1)\ast (1-Se2),-\,Se1\ast Se2),\\ \,\,\,\,\,\,min(Se1\ast (1-Se2),Se1\ast (1-Se2)))\end{array}$$

and

$$\begin{array}{c}{{\rm{covN}}}_{12} \sim Uniform\,(max(-(1-Sp1)\ast (1-Sp2),-\,Sp1\ast Sp2),\\ \,\,\,\,\,\,min(Sp1\ast (1-Sp2),Sp1\ast (1-Sp2)))\end{array}$$

We ran two simulation chains of 100,000 iterations whose convergence and mixing were assessed by checking the trace plots for all monitored parameters and calculating the Gelman-Rubin convergence statistics^36^. The first 5,000 iterations were discarded to allow for burn-in of the chains and the chains were thinned, taking every hundredth sample to reduce autocorrelation amongst the samples. For both H5N1 and H5N6 subtypes, significant pair-wise interactions were selected following a stepwise forward selection procedure using the Deviance Information Criterion (DIC). The DIC is based on a trade-off between the fit and the complexity of the model. It is generally accepted that models with smaller DIC are better supported by the data. The best model was considered to be the most parsimonious model whose DIC was less than two points greater than that of the model associated with the smallest DIC^37^.

## Results

A total of 15,180 individual samples were collected during the study period, generating 3036 (pooled-by-five) samples that were tested for the presence of H5N1 and H5N6 RNA. Most visits occurred in November and December 2014 (39% of the visits) and July and August 2015 (55%). After discarding consecutive visits happening less than 10 days apart from each other, the LBM survey resulted in 230 visits (88%) in which the desired sample size was achieved (*i.e.* 12 pooled samples were collected as described in Table 1), with 43, 46, 21, 3 and 4 LBMs having been visited once, twice, three times, four times and five times across the study period, respectively. Amongst the 230 visits, 26 (11%) and 43 (19%) resulted in a positive test result in at least one of the five sampling protocols for H5N1 and H5N6 subtypes, respectively. Five visits (2%) resulted in positive test results for both subtypes, suggesting that the apparent contamination status of a LBM for H5N1 was independent of that of H5N6 (χ^2^ < 10^−3^, df = 1, p > 0.05). Amongst the 74 LBMs that have been visited at least twice, 12 and 20 were tested positive multiple times for H5N1 and H5N6 subtypes, respectively. For both subtypes, the duck sampling protocol detected most of these positive markets, as 59% (respectively 87%) of the LBMs that were tested positive for H5N1 (resp. H5N6) had at least one out of six oropharyngeal duck samples that tested positive for H5N1 (resp. H5N6). The observed frequency of the 2^5^ = 32 different combinations of test results is given in Table 2.

The latent class model that best fitted the H5N1 data did not include any interaction between sampling protocols. However, the model that best fitted the H5N6 data included a positive interaction between the liquid waste and the drinking water sampling protocols in non-contaminated LBMs. This suggests that non-contaminated LBMs that tested negative (resp. positive) for H5N6 through the liquid waste protocol were more likely to also test negative (resp. positive) through the drinking water sampling protocol than those which tested positive (resp. negative). As illustrated by the trace plots of the parameters associated with the two final models (Supplementary Figures S1 and S2) and the Gelman-Rubin convergence diagnostic being 1 for all parameters, the two chains have converged and mixed satisfactorily for both subtypes.

**Figure 2:**
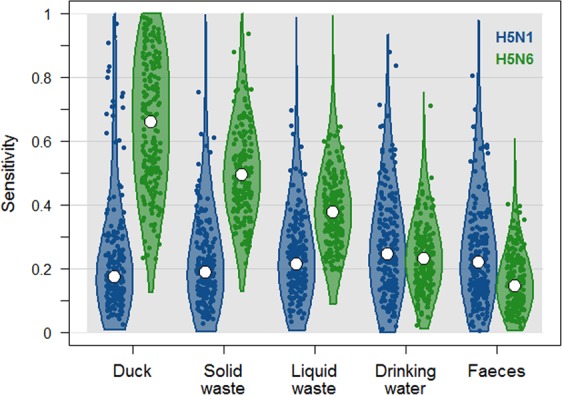
Posterior density of the sensitivity at the individual (pooled-by-five) sample level of the five different sample types for detecting H5N1 virus (in blue) and H5N6 virus (in green) in live-bird markets in Vietnam, as estimated by the best-fit models. White dots represent the median of the posterior distributions while the filled dot clouds and the violin plots illustrate the variability of the posterior distributions.

Figure 2 presents the sensitivity of each of the five sample types for both H5N1 and H5N6 subtypes. For the detection of H5N1 viruses, all sample types showed comparable performances with an estimated sensitivity ranging from 0.17 (95%CrI: 0.06–0.86) for the oropharyngeal duck samples to 0.25 (95%CrI: 0.04–0.63) for the drinking water. All five sensitivity estimates were associated with wide and overlapping confidence intervals. All sample types were associated with a H5N1 specificity that was more than 95% likely to be greater than 0.97 (Table 3). The model predicted that the proportion of LBMs that tested positive (apparent prevalence) for H5N1 was 0.13 (95%CrI: 0.09–0.18) while the proportion of LBMs truly contaminated (true prevalence) by H5N1 viruses was 0.05 (95%CrI: 0.01–0.13).

**Table 3:** Parameter estimates of the best-fit models for H5N1 and H5N6.

	H5N1		H5N6	
	Point estimate	95% CrI	Point estimate	95% CrI
Se duck sample	0.17	0.06–0.86	0.66	0.27–0.98
Sp duck sample	0.99	0.99–1.00	0.98	0.98–0.99
Se solid waste	0.19	0.05–0.59	0.49	0.27–0.81
Sp solid waste	1	0.99–1.00	1	0.99–1.00
Se liquid waste	0.21	0.06–0.53	0.38	0.20–0.60
Sp liquid waste	0.99	0.97–1.00	0.99	0.98–1.00
Se drinking water	0.25	0.04–0.63	0.23	0.08–0.46
Sp drinking water	1	0.99–1.00	0.99	0.98–1.00
Se faeces	0.22	0.04–0.64	0.14	0.03–0.35
Sp faeces	1	0.98–1.00	0.99	0.98–1.00
True prevalence	0.05	0.01–0.13	0.08	0.04–0.13
Apparent prevalence	0.13	0.09–0.18	0.20	0.15–0.25
CovN	—	—	0.003	0.000–0.015
DIC	58.5		61.2	

Estimated sensitivity (Se) and specificity (Sp) refer to the individual (pooled-by-five) samples of the different sample types; the prevalence refers to the proportion of contaminated live-bird markets (LBMs); 95%CrI is the 95% credible interval of the posterior distribution of the parameters, as estimated by the best-fit models; CovN is the estimated covariance between liquid waste and drinking water samples in non-contaminated LBMs; DIC stands for deviance information criterion. Note that the two selected models were those associated with the smallest DIC.

For H5N6, oropharyngeal duck samples appeared to be the most effective sampling strategy, with an estimated sensitivity reaching 0.66 (95%CrI: 0.27–0.98). It was followed by the solid waste samples (Se = 0.49; 95%CrI: 0.27–0.81) and the liquid waste samples (Se = 0.38; 95%: 0.20–0.60). Faecal samples were associated with the lowest sensitivity, estimated at 0.14 (95%CI: 0.03–0.35). All sample types showed a H5N6 specificity that was more than 95% likely to be greater than 0.97. Similarly to H5N1, the model predicted that the proportion of LBM that tested positive (apparent prevalence) for H5N6 was 0.20 (95%CI: 0.15–0.25) while the proportion of LBM truly contaminated (true prevalence) by H5N6 viruses was 0.08 (95%CI: 0.04–0.13).

**Figure 3:**
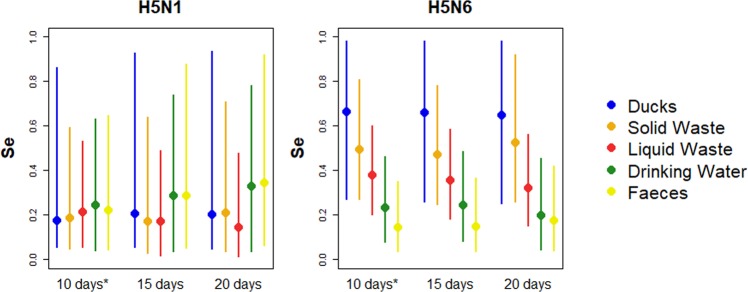
Results of the sensitivity analysis for the H5N1 model (left) and the H5N6 model (right). Coloured dots and bars represent respectively the median and the 95% credible interval of the posterior distribution of the sensitivity at the sample level of the five different sample types in live-bird markets in Vietnam, as estimated by the best-fit models. On the x-axis, 10, 15 and 20 days represent the minimum timelag that is assumed in order to consider two successive visits of the same market as independent. The wildcard (*) represents the baseline assumption used in this study.

Figure 3 illustrates the results of the sensitivity analysis by representing the estimated sensitivity of each sample type for each of the three assumptions (minimum timelag of 10, 15 or 20 days between successive visits of the same market). The results of the best-fit H5N1 model appear sensitive to the assumption since the ranking of the sample types according to their most likely effectiveness for detecting H5N1 varies according to the assumption, even though the 95% credible intervals remain largely overlapping. On the contrary, the results of the best-fit H5N6 model present a good robustness to the assumption, since the rankings are maintained and the 95% credible intervals not substantially altered.

By combining the estimated performance of the five different sample types according to the sampling strategy that was used to collect the data presented in this study (Table 1), we evaluated the posterior distributions of the sensitivity and specificity of the overall sampling strategy for H5N1 and H5N6 subtypes. As supported by the data, we are 95% confident that the sensitivity, *i.e.* the probability that at least one of the 12 samples would test positive in a LBM contaminated by H5N1 subtype (resp. H5N6 subtype), is greater than 78% (resp. 99%). Similarly, we are 95% confident that the specificity, *i.e.* the probability that all 12 samples would test negative in a H5N1-free (resp. H5N6-free) LBM, lays between 86% and 97% (resp. between 82% and 92%), explaining the expected presence of false positives for both subtypes.

**Figure 4:**
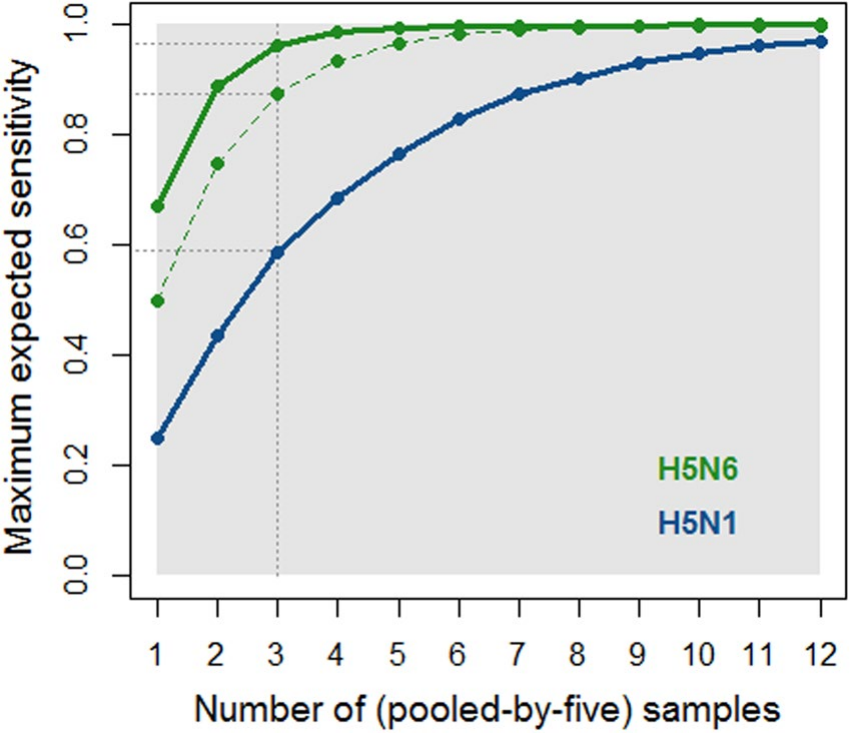
Maximum sensitivity of detection that could be expected amongst all combinations of sampling strategies involving an increasing number of samples (from 1 to 12) for H5N1 viruses (in blue) and H5N6 viruses (in green). For a given number of samples, all combinations of the five sample types are considered. The solid lines refer to sampling strategies that do not exclude oropharyngeal duck samples while the coloured dashed lines refer to sampling strategies that involve combinations of environmental samples only, thus excluding oropharyngeal duck samples. Note that for H5N1 viruses, solid and dashed lines are superimposed. The y-axis represents the median of the posterior distribution of the sensitivity of the most sensitive sampling strategy for a given number of samples. As an example (see grey dotted lines), for the collection of three (pooled-by-five) samples, the most sensitive sampling strategies that do not exclude oropharyngeal duck samples have an expected sensitivity of 0.59 and 0.97 for H5N1 and H5N6, respectively. Excluding oropharyngeal duck samples does not affect the sensitivity for H5N1, but decreases the maximum expected sensitivity for H5N6 to 0.88.

Finally, using the model outputs, we simulated all combinations of sample types in order to identify parsimonious sampling strategies that ensured high sensitivity for both H5N1 and H5N6 subtypes. As shown in Fig. 4, detecting H5N1- or H5N6-contaminated LBMs in Vietnam does not require the same sampling effort, with H5N1 viruses being the most difficult to detect. As an example, considering the collection of three samples of any kind, the sensitivity of the most sensitive sampling strategy for H5N1 would be around 59% whether or not oropharyngeal duck samples are included. On the other hand, the most sensitive sampling strategy for H5N6 involving only environmental samples would be around 88%, while the sensitivity of the most sensitive sampling strategy including oropharyngeal duck samples would be around 97% (Fig. 4).

As described earlier in that section, 78% (respectively 99%) correspond to the 5^th^ percentile of the posterior distribution of the sensitivity of the current sampling strategy for H5N1 (resp. H5N6), as estimated by the best-fit model for H5N1 (resp. H5N6). We identified 42 more parsimonious (*i.e.* with fewer than 12 samples) sampling strategies associated with a sensitivity for H5N1 that had 95% chance to be greater than 78%, none of which involved the collection of environmental samples only; for H5N6, we identified 1998 (resp. 94) sampling strategies associated with a sensitivity that had 95% chance to be greater than 95% (resp. 99%), of which 235 (resp. 0) involved the collection of environmental samples only.

Being 95% confident that the sensitivities of a given sampling strategy for detecting H5N1 and H5N6 subtypes in contaminated markets are respectively greater than 78% and 95% can be achieved by 42 different sampling strategies more parsimonious than the current strategy, all of which involved the collection of oropharyngeal duck samples (SuppMat). With 12 (pooled-by-five) samples to be collected, we identified 27 sampling strategies involving the collection of environmental samples only that lead to sensitivities that have 95% chance to be greater than 78% for H5N1 and 95% for H5N6 (SuppMat). As an example, collecting 2 (pooled-by-five) solid waste samples, 5 liquid waste samples, 2 drinking water samples and 3 faeces samples would allow to be 95% confident that the overall sensitivity and specificity are greater than 80% and 96%, respectively.

## Discussion

Optimising surveillance designs by focusing on sample materials that are more likely to test positive in contaminated LBMs is of primary importance in the context of limited budget allocated to surveillance, particularly in low- and middle-income countries like Vietnam. In agreement with previous work^8,10,18^, this study provided evidence that the detectability of avian influenza viruses in contaminated LBMs is highly dependent on the type of sample collected and on the circulating subtype. It demonstrated that the sensitivity of environmental samples for detecting H5N1 viruses in LBMs is equivalent to that of oropharyngeal duck samples, as previously suggested in Indonesia^10^; however, taking oropharyngeal duck samples is expected to be more effective in detecting H5N6 viruses than taking any of the four environmental samples. Also, despite the specificity of the individual (pooled-by-five) samples of the different sample types having been found to be very similar and close to 1, collecting 12 samples per market likely led to a sub-optimal specificity of the overall surveillance strategy in LBMs and consequently to a significant number of false positive LBMs, both for H5N1 and H5N6 subtypes. Finally, we identified a range of sampling strategies that are of similar expected sensitivity to the current sampling protocol but that involve fewer samples (leading to a greater expected specificity and smaller surveillance expenditure) or even no oropharyngeal duck samples (leading to reduced efforts in the field and greater acceptability by poultry sellers). The details of these optimised sampling strategies are given as a Supplementary Table.

Sampling live birds has become controversial as sampling teams often consider it to be impractical and farmers or traders as too invasive into their business operation^21^. As an alternative, environmental samples are easier to collect, less time-consuming and do not interfere with trading or selling activities. While environmental samples were found to be as effective as oropharyngeal samples at detecting H5N1 viruses in contaminated LBMs making them an obvious alternative to live bird sampling, they do not seem to perform as well for H5N6 viruses. However, apart from the faeces samples, their expected effectiveness at detecting H5N6 viruses is systematically equal to or greater than their effectiveness at detecting H5N1 viruses (Fig. 2). Therefore, if the objective of the sampling protocol is to detect both subtypes, a satisfactory overall sensitivity can be achieved by collecting only environmental samples (Supplementary Table).

Faeces were shown to be the least sensitive sample type (of the five tested) for detecting H5N6 virus in contaminated LBMs while, for the same viral subtype, oropharyngeal duck samples appeared to be the most sensitive strategy (Fig. 2, Table 3). This pattern could suggest that the HPAI H5N6 strains that circulated in Vietnamese LBMs in 2014–2015 were more likely to replicate in and be excreted by the respiratory than the digestive tract, which is consistent with observations from experimental infections conducted in China using a H5N6 strain isolated in 2014^38^. Similarly, model results also suggest that this tropism was much less clear-cut for the detected Vietnamese H5N1 strains. The sensitivities for H5N6 being generally greater than those for H5N1 (Fig. 2) tends to suggest that the level of contamination was higher in H5N6-contaminated LBMs than in H5N1-contaminated LBMs.

The rRT-PCR technique that was used to identify contaminated markets is able to detect small fragments of viral RNA, even if the viruses are already inactivated. Indeed, the type of organic matter, the level of humidity, the temperature, the pH, and other environmental parameters are likely to affect virus survival outside the host^39,40^. Therefore, a positive environmental sample is less likely to be useful for virus isolation or viral sequencing than a positive sample directly collected on live birds. As a consequence, the implications of this study’s results for surveillance design need to be considered in the context of the objectives of the surveillance activities. Focusing on environmental samples would be relevant for surveillance objectives related to the monitoring of the spatio-temporal dynamic of circulating subtypes and the implementation of intervention measures in positive markets; however, this strategy would probably be of limited relevance for surveillance objectives related to molecular characterisation of circulating strains and estimation of prevalence or force of infection within LBMs.

Since only a relatively small number of LBMs tested positive for H5N1 or H5N6 viruses (26 and 43 out of 230, respectively), a large number of test result combinations were associated with zero observations (20 and 19 out of 32, respectively). This inherent constraint of the available data led to relatively wide and largely overlapping credible intervals around the point estimates of the sensitivity parameters (particularly for H5N1), making it difficult to draw unequivocal conclusions as to which sample type is the most sensitive for detecting H5N1 and H5N6 viruses. Before producing recommendations regarding which sample type to collect in LBMs, similar studies would be needed in other contexts (different time period or different location). Despite this limitation, it is worth stressing that the 95% credible interval of the posterior distribution of the frequency of all 32 combinations of test results contain the observed frequency (Table 2), demonstrating that the models satisfactorily captured the information contained in the data. The lower number of H5N1-positive markets also made the H5N1 model much more sensitive than the H5N6 model to the assumption of a minimum timelag of 10 days for considering two successive visits of the same market as independent.

This latent-class model assumes that all markets have the same probability of being contaminated and all contaminated markets have the same level of contamination. However, LBMs may not be equivalent in terms of avian influenza risk^41^. Gilbert *et al*.^42^ showed that the risk of H7N9 in China increased with increasing LBM density and increasing density of intensively-raised chickens. In addition, Wang *et al*.^24^ showed that LBMs with longer duration of sales per day and a large number of poultry types were associated with an increased risk of H5, H7 or H9 avian influenza virus presence. To strengthen the analysis and qualify the inference, such study should ideally consider different populations of LBMs depending on their risk of contamination. This would allow the estimation of the between-LBM prevalence in the different risk populations and a more accurate estimation of the sensitivity and specificity parameters. However, market-level variables potentially associated with the risk of AIV contamination were not systematically collected at the time of sampling, preventing refining the estimations presented in this study.

By using dichotomous test result outcomes relating to both environmental and live-bird sample types, the model is able to compare the effectiveness of the different sample protocols but cannot explicitly account for the prevalence or the level of contamination within LBMs. These latter parameters are implicitely included into the sensitivity estimates: a sampling protocol implemented in heavily contaminated LBMs would be associated with larger sensitivity estimates than the same sampling protocol implemented in lightly contaminated LBMs. More generally, the estimated sensitivities of the different sample types refer to the context of the H5N1 and H5N6 viruses that circulated in Vietnamese LBMs in 2014 and 2015. Indeed, the estimated higher sensitivities for H5N6 can be the consequence of a larger within-LBM contamination with H5N6 compared to H5N1 strains. Consequently, a different context with different contamination levels is likely to generate different sensitivity estimates. Similarly, different strains, even from the same subtype, with different shedding patterns are also likely to lead to different estimates.

It is acknowledged that cloacal samples and chicken samples were lacking in this study, preventing the comparison of all possible sampling strategies. Similarly, the study did not consider samples collected in slaughter and dressing areas, even though these settings have been shown to be excellent sites for AIV detection^10,43^. This is because many markets in Vietnam do not have these facilities preventing the collection of a standard set of samples from all markets. Notwithstanding, this study generated results that are crucial for making an evidence-based decision when it comes to surveillance design and provided quantitative evidence of the relevance of environmental samples for monitoring H5N1 viruses. It also presented a robust and flexible statistical modelling approach that should be applied to other contexts in order to advance our collective understanding of how HPAI surveillance should be designed in LBMs.

## Supplementary information

Supplementary Materials

## Data Availability

All data necessary to reproduce the results generated by this study is available in Table 2.
