# Supplementary material for: Optimising the detectability of H5N1 and H5N6 highly pathogenic avian influenza viruses in Vietnamese live-bird markets
Source: Sci Rep. 2019 Jan 31;9:1031. doi: 10.1038/s41598-018-37616-1 (PMC6355762; doi:10.1038/s41598-018-37616-1)
Supplement: Supplementary file 1 — Supplementary Materials [file 41598_2018_37616_MOESM1_ESM.docx]

**SUPPLEMENTARY MATERIALS**

# **Optimising the detectability of H5N1 and H5N6 highly pathogenic avian influenza viruses in Vietnamese live-bird markets**

**Timothée Vergne^1,2,3^*, Anne Meyer^1^, Pham Thanh Long^4^, Doaa A. Elkholly^1^, Ken Inui^5^, Pawin Padungtod^5^, Scott H. Newman^5^, Guillaume Fournié^1^, Dirk U. Pfeiffer^1,6^**

*^1^ VEEPH Group, Royal Veterinary College, Hatfield, United Kingdom*

*^2^ MIVEGEC Group, Institut de Recherche pour le Développement, Montpellier, France*

*^3^ UMR IHAP, University of Toulouse, INRA, ENVT, Toulouse, France*

*^4^ Department of Animal Health, Ministry of Agriculture and Rural Development, Hanoi, Vietnam*

*^5^ Food and Agriculture Organization of the United Nations, Hanoi, Vietnam*

*^6^ Centre for Applied One Health Research and Policy Advice, City University of Hong-Kong, Hong-Kong SAR, PR China*

## Supplementary method SM1: WinBUGS script of the model accounting for an interaction between the sampling protocols 1 and 2

model;

{

y1[1:32] ~ dmulti(p1[1:32], n1)

p1[1]<-prev1*((1-Se1)*(1-Se2))*((1-Se3)*(1-Se4))*(1-Se5)+(1-prev1)*(Sp1*Sp2+covN)*(Sp3*Sp4)*Sp5

p1[2]<-prev1*(Se1*(1-Se2))*((1-Se3)*(1-Se4))*(1-Se5)+(1-prev1)*((1-Sp1)*Sp2-covN)*(Sp3*Sp4)*Sp5

p1[3]<-prev1*((1-Se1)*Se2)*((1-Se3)*(1-Se4))*(1-Se5)+(1-prev1)*(Sp1*(1-Sp2)-covN)*(Sp3*Sp4)*Sp5

p1[4]<-prev1*((1-Se1)*(1-Se2))*(Se3*(1-Se4))*(1-Se5)+(1-prev1)*(Sp1*Sp2+covN)*((1-Sp3)*Sp4)*Sp5

p1[5]<-prev1*((1-Se1)*(1-Se2))*((1-Se3)*Se4)*(1-Se5)+(1-prev1)*(Sp1*Sp2+covN)*(Sp3*(1-Sp4))*Sp5

p1[6]<-prev1*((1-Se1)*(1-Se2))*((1-Se3)*(1-Se4))*Se5+(1-prev1)*(Sp1*Sp2+covN)*(Sp3*Sp4)*(1-Sp5)

p1[7]<-prev1*(Se1*Se2)*((1-Se3)*(1-Se4))*(1-Se5)+(1-prev1)*((1-Sp1)*(1-Sp2)+covN)*(Sp3*Sp4)*Sp5

p1[8]<-prev1*(Se1*(1-Se2))*(Se3*(1-Se4))*(1-Se5)+(1-prev1)*((1-Sp1)*Sp2-covN)*((1-Sp3)*Sp4)*Sp5

p1[9]<-prev1*(Se1*(1-Se2))*((1-Se3)*Se4)*(1-Se5)+(1-prev1)*((1-Sp1)*Sp2-covN)*(Sp3*(1-Sp4))*Sp5

p1[10]<-prev1*(Se1*(1-Se2))*((1-Se3)*(1-Se4))*Se5+(1-prev1)*((1-Sp1)*Sp2-covN)*(Sp3*Sp4)*(1-Sp5)

p1[11]<-prev1*((1-Se1)*Se2)*(Se3*(1-Se4))*(1-Se5)+(1-prev1)*(Sp1*(1-Sp2)-covN)*((1-Sp3)*Sp4)*Sp5

p1[12]<-prev1*((1-Se1)*Se2)*((1-Se3)*Se4)*(1-Se5)+(1-prev1)*(Sp1*(1-Sp2)-covN)*(Sp3*(1-Sp4))*Sp5

p1[13]<-prev1*((1-Se1)*Se2)*((1-Se3)*(1-Se4))*Se5+(1-prev1)*(Sp1*(1-Sp2)-covN)*(Sp3*Sp4)*(1-Sp5)

p1[14]<-prev1*((1-Se1)*(1-Se2))*(Se3*Se4)*(1-Se5)+(1-prev1)*(Sp1*Sp2+covN)*((1-Sp3)*(1-Sp4))*Sp5

p1[15]<-prev1*((1-Se1)*(1-Se2))*(Se3*(1-Se4))*Se5+(1-prev1)*(Sp1*Sp2+covN)*((1-Sp3)*Sp4)*(1-Sp5)

p1[16]<-prev1*((1-Se1)*(1-Se2))*((1-Se3)*Se4)*Se5+(1-prev1)*(Sp1*Sp2+covN)*(Sp3*(1-Sp4))*(1-Sp5)

p1[17]<-prev1*(Se1*Se2)*(Se3*(1-Se4))*(1-Se5)+(1-prev1)*((1-Sp1)*(1-Sp2)+covN)*((1-Sp3)*Sp4)*Sp5

p1[18]<-prev1*(Se1*Se2)*((1-Se3)*Se4)*(1-Se5)+(1-prev1)*((1-Sp1)*(1-Sp2)+covN)*(Sp3*(1-Sp4))*Sp5

p1[19]<-prev1*(Se1*Se2)*((1-Se3)*(1-Se4))*Se5+(1-prev1)*((1-Sp1)*(1-Sp2)+covN)*(Sp3*Sp4)*(1-Sp5)

p1[20]<-prev1*(Se1*(1-Se2))*(Se3*Se4)*(1-Se5)+(1-prev1)*((1-Sp1)*Sp2-covN)*((1-Sp3)*(1-Sp4))*Sp5

p1[21]<-prev1*(Se1*(1-Se2))*(Se3*(1-Se4))*Se5+(1-prev1)*((1-Sp1)*Sp2-covN)*((1-Sp3)*Sp4)*(1-Sp5)

p1[22]<-prev1*(Se1*(1-Se2))*((1-Se3)*Se4)*Se5+(1-prev1)*((1-Sp1)*Sp2-covN)*(Sp3*(1-Sp4))*(1-Sp5)

p1[23]<-prev1*((1-Se1)*Se2)*(Se3*Se4)*(1-Se5)+(1-prev1)*(Sp1*(1-Sp2)-covN)*((1-Sp3)*(1-Sp4))*Sp5

p1[24]<-prev1*((1-Se1)*Se2)*(Se3*(1-Se4))*Se5+(1-prev1)*(Sp1*(1-Sp2)-covN)*((1-Sp3)*Sp4)*(1-Sp5)

p1[25]<-prev1*((1-Se1)*Se2)*((1-Se3)*Se4)*Se5+(1-prev1)*(Sp1*(1-Sp2)-covN)*(Sp3*(1-Sp4))*(1-Sp5)

p1[26]<-prev1*((1-Se1)*(1-Se2))*(Se3*Se4)*Se5+(1-prev1)*(Sp1*Sp2+covN)*((1-Sp3)*(1-Sp4))*(1-Sp5)

p1[27]<-prev1*(Se1*Se2)*(Se3*Se4)*(1-Se5)+(1-prev1)*((1-Sp1)*(1-Sp2)+covN)*((1-Sp3)*(1-Sp4))*Sp5

p1[28]<-prev1*(Se1*Se2)*(Se3*(1-Se4))*Se5+(1-prev1)*((1-Sp1)*(1-Sp2)+covN)*((1-Sp3)*Sp4)*(1-Sp5)

p1[29]<-prev1*(Se1*Se2)*((1-Se3)*Se4)*Se5+(1-prev1)*((1-Sp1)*(1-Sp2)+covN)*(Sp3*(1-Sp4))*(1-Sp5)

p1[30]<-prev1*(Se1*(1-Se2))*(Se3*Se4)*Se5+(1-prev1)*((1-Sp1)*Sp2-covN)*((1-Sp3)*(1-Sp4))*(1-Sp5)

p1[31]<-prev1*((1-Se1)*Se2)*(Se3*Se4)*Se5+(1-prev1)*(Sp1*(1-Sp2)-covN)*((1-Sp3)*(1-Sp4))*(1-Sp5)

p1[32]<-prev1*(Se1*Se2)*(Se3*Se4)*Se5+(1-prev1)*((1-Sp1)*(1-Sp2)+covN)*((1-Sp3)*(1-Sp4))*(1-Sp5)

prev1 ~ dbeta(1,1)

#Ducks

Se.duck ~ dbeta(1,1)

Sp.duck ~ dbeta(1,1)

complement.Se.duck <-1-Se.duck

Se1<-1-pow(complement.Se.duck,6)

Sp1<-pow(Sp.duck ,6)

#Solid waste

Se.SW ~ dbeta(1,1)

Sp.SW ~ dbeta(1,1)

complement.Se.SW <-1-Se.SW

Se2<-1-pow(complement.Se.SW,2)

Sp2<-pow(Sp.SW,2)

#Liquid waste

Se.LW ~ dbeta(1,1)

Sp.LW ~ dbeta(1,1)

complement.Se.LW<-1-Se.LW

Se3<-1-pow(complement.Se.LW,2)

Sp3<-pow(Sp.LW,2)

#Drinking water

Se.DW ~ dbeta(1,1)

Sp.DW ~ dbeta(1,1)

Se4<- Se.DW

Sp4<- Sp.DW

#Faeces

Se.F ~ dbeta(1,1)

Sp.F ~ dbeta(1,1)

Se5<-Se.F

Sp5<- Sp.F

#Dependence term between tests 1 and 2 in non-contaminated markets

covN ~ dunif(lc1, uc1)

lc1 <- max(-(1-Sp2)*(1-Sp1),-Sp2*Sp1)

uc1 <- min(Sp2*(1-Sp1),Sp1*(1-Sp2))

rhoN <- covN / sqrt(Sp1*(1-Sp1)*Sp2*(1-Sp2))

}

## Supplementary Figure S1: Trace plots of the parameters associated with the final H5N1 model


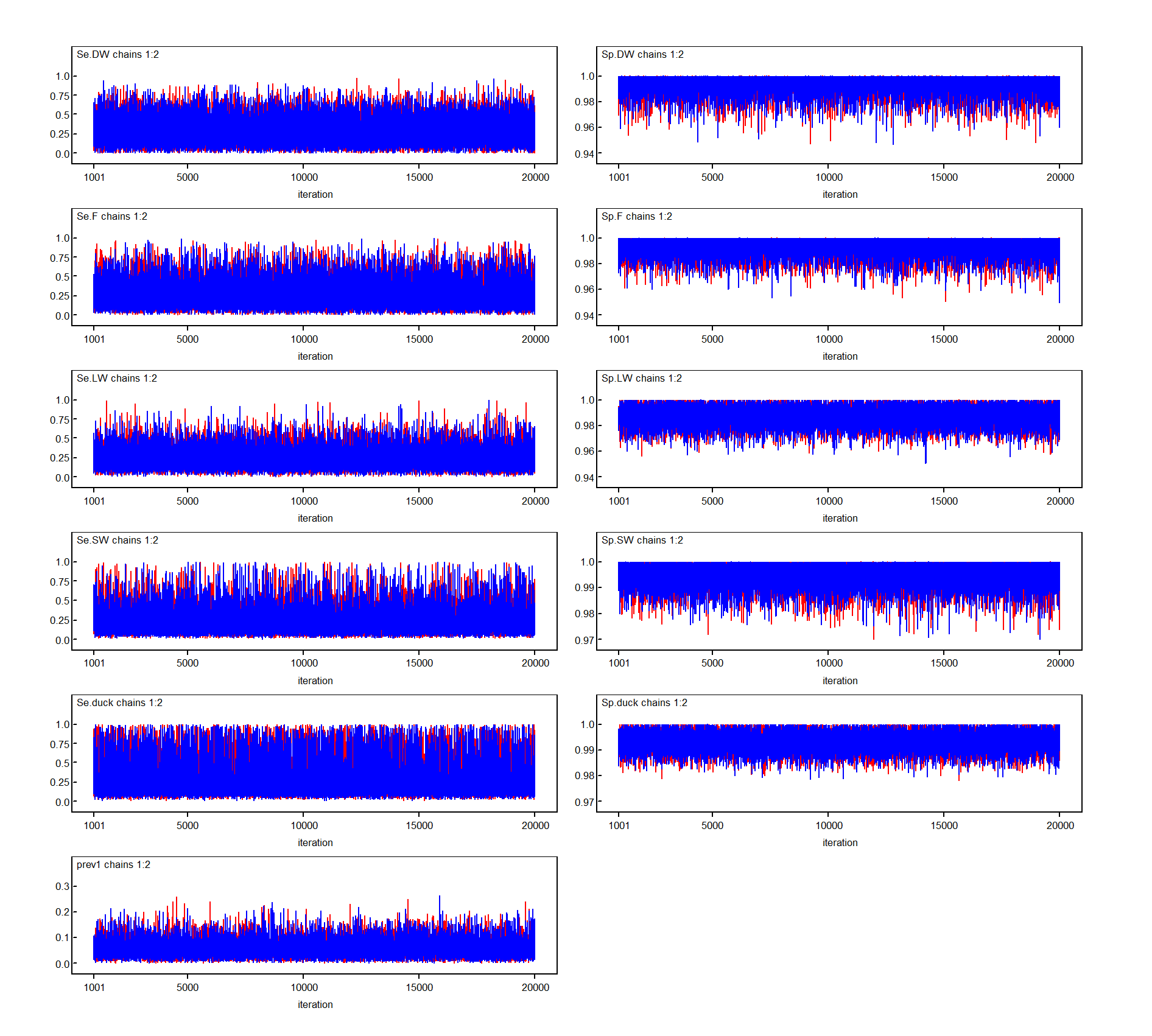


## Supplementary Figure S2: Trace plots of the parameters associated with the final H5N6 model

##
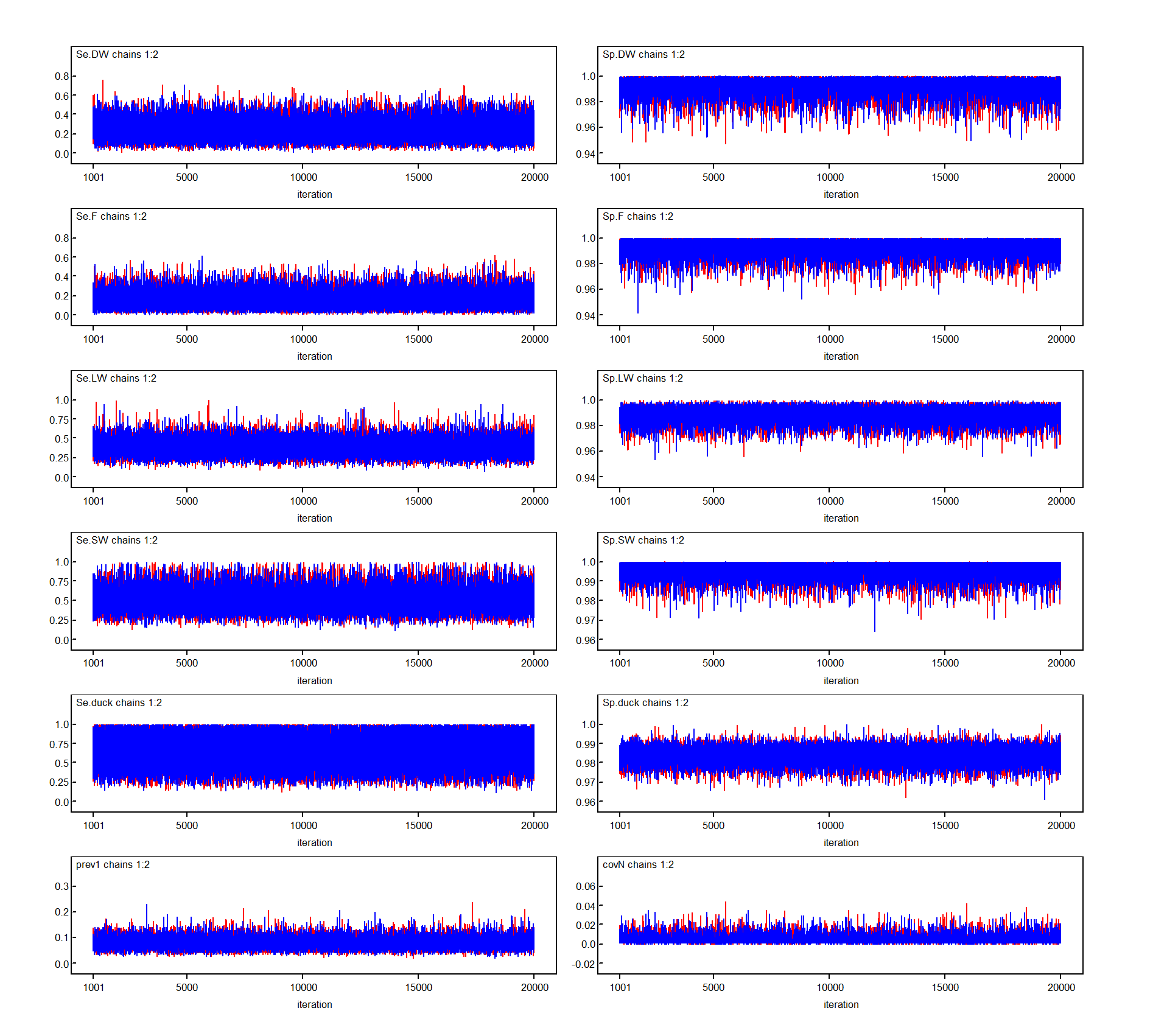


## Supplementary Table S1: More parsimonious sampling strategies associated with a sensitivity equivalent to the strategy that was used to collect the data. Se.H5N1 and Se.H5N6 stand for the median of the posterior distribution of the overall sensitivity of the sampling strategy for detecting H5N1 and H5N6, respectively.

| **Number of (pooled-by-five) samples collected of each type** | | | | |  |  |
| --- | --- | --- | --- | --- | --- | --- |
| **Duck** | **Solid waste** | **Liquid waste** | **Drinking water** | **Faeces** | **Se.H5N1** | **Se.H5N6** |
| 4 | 1 | 3 | 2 | 1 | 0.783 | 0.982 |
| 4 | 1 | 2 | 3 | 1 | 0.780 | 0.979 |
| 4 | 0 | 4 | 2 | 1 | 0.784 | 0.979 |
| 3 | 2 | 3 | 2 | 1 | 0.781 | 0.985 |
| 3 | 1 | 4 | 2 | 1 | 0.790 | 0.984 |
| 3 | 1 | 4 | 1 | 2 | 0.783 | 0.981 |
| 3 | 1 | 3 | 3 | 1 | 0.782 | 0.981 |
| 3 | 1 | 3 | 2 | 2 | 0.782 | 0.979 |
| 3 | 1 | 2 | 4 | 1 | 0.781 | 0.977 |
| 3 | 1 | 2 | 3 | 2 | 0.784 | 0.975 |
| 3 | 0 | 5 | 2 | 1 | 0.780 | 0.978 |
| 3 | 0 | 4 | 3 | 1 | 0.784 | 0.975 |
| 3 | 0 | 4 | 2 | 2 | 0.784 | 0.973 |
| 3 | 0 | 3 | 3 | 2 | 0.788 | 0.968 |
| 3 | 0 | 3 | 2 | 3 | 0.780 | 0.965 |
| 3 | 0 | 2 | 4 | 2 | 0.780 | 0.960 |
| 2 | 2 | 4 | 2 | 1 | 0.780 | 0.984 |
| 2 | 2 | 3 | 3 | 1 | 0.781 | 0.982 |
| 2 | 2 | 3 | 2 | 2 | 0.782 | 0.979 |
| 2 | 2 | 2 | 4 | 1 | 0.782 | 0.978 |
| 2 | 2 | 2 | 3 | 2 | 0.782 | 0.977 |
| 2 | 1 | 5 | 2 | 1 | 0.784 | 0.981 |
| 2 | 1 | 5 | 1 | 2 | 0.781 | 0.979 |
| 2 | 1 | 4 | 3 | 1 | 0.788 | 0.979 |
| 2 | 1 | 4 | 2 | 2 | 0.791 | 0.976 |
| 2 | 1 | 3 | 4 | 1 | 0.781 | 0.975 |
| 2 | 1 | 3 | 3 | 2 | 0.787 | 0.972 |
| 2 | 1 | 3 | 2 | 3 | 0.782 | 0.970 |
| 2 | 1 | 2 | 4 | 2 | 0.783 | 0.968 |
| 2 | 0 | 5 | 3 | 1 | 0.784 | 0.972 |
| 2 | 0 | 5 | 2 | 2 | 0.788 | 0.969 |
| 2 | 0 | 4 | 3 | 2 | 0.786 | 0.965 |
| 2 | 0 | 4 | 2 | 3 | 0.782 | 0.960 |
| 2 | 0 | 3 | 4 | 2 | 0.785 | 0.958 |
| 2 | 0 | 3 | 3 | 3 | 0.782 | 0.955 |
| 2 | 0 | 3 | 2 | 4 | 0.780 | 0.951 |
| 1 | 1 | 5 | 2 | 2 | 0.782 | 0.971 |
| 1 | 1 | 4 | 3 | 2 | 0.780 | 0.967 |
| 1 | 1 | 4 | 2 | 3 | 0.780 | 0.965 |
| 1 | 0 | 5 | 3 | 2 | 0.783 | 0.955 |
| 1 | 0 | 5 | 2 | 3 | 0.783 | 0.952 |
| 1 | 0 | 4 | 4 | 2 | 0.780 | 0.951 |

## Supplementary Table S2: Environmental-sample-only strategies associated with a sensitivity equivalent to the strategy that was used to collect the data. Se.H5N1 and Se.H5N6 stand for the median of the posterior distribution of the overall sensitivity of the sampling strategy for detecting H5N1 and H5N6, respectively. Note that the strategy highlighted in grey corresponds to the one cited in the manuscript.

| **Number of (pooled-by-five) samples collected of each type** | | | | |  |  |  |
| --- | --- | --- | --- | --- | --- | --- | --- |
| **Duck** | **Solid waste** | **Liquid waste** | **Drinking water** | **Faeces** | **Se.H5N1** | **Se.H5N6** |  |
| 0 | 4 | 5 | 2 | 1 | 0.780 | 0.979 |  |
| 0 | 4 | 4 | 2 | 2 | 0.781 | 0.975 |  |
| 0 | 3 | 6 | 1 | 2 | 0.783 | 0.974 |  |
| 0 | 3 | 5 | 3 | 1 | 0.780 | 0.973 |  |
| 0 | 3 | 5 | 2 | 2 | 0.795 | 0.973 |  |
| 0 | 3 | 5 | 1 | 3 | 0.789 | 0.969 |  |
| 0 | 3 | 4 | 3 | 2 | 0.788 | 0.969 |  |
| 0 | 3 | 4 | 2 | 3 | 0.789 | 0.967 |  |
| 0 | 3 | 4 | 1 | 4 | 0.781 | 0.961 |  |
| 0 | 3 | 3 | 4 | 2 | 0.783 | 0.964 |  |
| 0 | 3 | 3 | 3 | 3 | 0.782 | 0.962 |  |
| 0 | 2 | 6 | 3 | 1 | 0.784 | 0.968 |  |
| 0 | 2 | 6 | 2 | 2 | 0.796 | 0.967 |  |
| 0 | 2 | 6 | 1 | 3 | 0.786 | 0.963 |  |
| 0 | 2 | 5 | 4 | 1 | 0.784 | 0.965 |  |
| 0 | 2 | 5 | 3 | 2 | 0.799 | 0.964 |  |
| 0 | 2 | 5 | 2 | 3 | 0.799 | 0.961 |  |
| 0 | 2 | 5 | 1 | 4 | 0.786 | 0.956 |  |
| 0 | 2 | 4 | 4 | 2 | 0.791 | 0.959 |  |
| 0 | 2 | 4 | 3 | 3 | 0.797 | 0.956 |  |
| 0 | 2 | 4 | 2 | 4 | 0.791 | 0.953 |  |
| 0 | 2 | 3 | 5 | 2 | 0.784 | 0.950 |  |
| 0 | 2 | 3 | 4 | 3 | 0.789 | 0.950 |  |
| 0 | 1 | 7 | 2 | 2 | 0.789 | 0.957 |  |
| 0 | 1 | 7 | 1 | 3 | 0.783 | 0.954 |  |
| 0 | 1 | 6 | 3 | 2 | 0.797 | 0.953 |  |
| 0 | 1 | 5 | 4 | 2 | 0.788 | 0.950 |  |

## Supplementary Table S3: Distribution of the 32 cross-classified results of the five sampling protocols for H5N1 and H5N6 subtypes, for the three different assumptions (10, 20 and 20 days) regarding the minimum timelag required for considering two successive visits as independent.

| **Sampling protocol** | | | | | **H5N1** | | | **H5N6** | | |
| --- | --- | --- | --- | --- | --- | --- | --- | --- | --- | --- |
| **Ducks** | **Solid waste** | **Liquid waste** | **Drinking water** | **Faeces** | **10 days** | **15 days** | **20 days** | **10 days** | **15 days** | **20 days** |
| 0 | 0 | 0 | 0 | 0 | 204 | 170 | 156 | 187 | 155 | 142 |
| 1 | 0 | 0 | 0 | 0 | 10 | 9 | 9 | 20 | 15 | 14 |
| 0 | 1 | 0 | 0 | 0 | 2 | 2 | 2 | 1 | 1 | 1 |
| 0 | 0 | 1 | 0 | 0 | 5 | 4 | 3 | 3 | 3 | 3 |
| 0 | 0 | 0 | 1 | 0 | 0 | 0 | 0 | 0 | 0 | 0 |
| 0 | 0 | 0 | 0 | 1 | 1 | 0 | 0 | 1 | 1 | 1 |
| 1 | 1 | 0 | 0 | 0 | 0 | 0 | 0 | 5 | 5 | 5 |
| 1 | 0 | 1 | 0 | 0 | 2 | 2 | 1 | 2 | 2 | 1 |
| 1 | 0 | 0 | 1 | 0 | 1 | 1 | 1 | 1 | 1 | 1 |
| 1 | 0 | 0 | 0 | 1 | 1 | 1 | 1 | 0 | 0 | 0 |
| 0 | 1 | 1 | 0 | 0 | 0 | 0 | 0 | 0 | 0 | 0 |
| 0 | 1 | 0 | 1 | 0 | 1 | 1 | 1 | 0 | 0 | 0 |
| 0 | 1 | 0 | 0 | 1 | 0 | 0 | 0 | 0 | 0 | 0 |
| 0 | 0 | 1 | 1 | 0 | 1 | 1 | 1 | 1 | 1 | 1 |
| 0 | 0 | 1 | 0 | 1 | 0 | 0 | 0 | 0 | 0 | 0 |
| 0 | 0 | 0 | 1 | 1 | 0 | 0 | 0 | 0 | 0 | 0 |
| 1 | 1 | 1 | 0 | 0 | 1 | 0 | 0 | 5 | 4 | 4 |
| 1 | 1 | 0 | 1 | 0 | 0 | 0 | 0 | 0 | 0 | 0 |
| 1 | 1 | 0 | 0 | 1 | 1 | 1 | 1 | 0 | 0 | 0 |
| 1 | 0 | 1 | 1 | 0 | 0 | 0 | 0 | 1 | 1 | 1 |
| 1 | 0 | 1 | 0 | 1 | 0 | 0 | 0 | 0 | 0 | 0 |
| 1 | 0 | 0 | 1 | 1 | 0 | 0 | 0 | 0 | 0 | 0 |
| 0 | 1 | 1 | 1 | 0 | 0 | 0 | 0 | 0 | 0 | 0 |
| 0 | 1 | 1 | 0 | 1 | 0 | 0 | 0 | 0 | 0 | 0 |
| 0 | 1 | 0 | 1 | 1 | 0 | 0 | 0 | 0 | 0 | 0 |
| 0 | 0 | 1 | 1 | 1 | 0 | 0 | 0 | 0 | 0 | 0 |
| 1 | 1 | 1 | 1 | 0 | 0 | 0 | 0 | 1 | 1 | 0 |
| 1 | 1 | 1 | 0 | 1 | 0 | 0 | 0 | 1 | 1 | 1 |
| 1 | 1 | 0 | 1 | 1 | 0 | 0 | 0 | 0 | 0 | 0 |
| 1 | 0 | 1 | 1 | 1 | 0 | 0 | 0 | 0 | 0 | 0 |
| 0 | 1 | 1 | 1 | 1 | 0 | 0 | 0 | 0 | 0 | 0 |
| 1 | 1 | 1 | 1 | 1 | 0 | 0 | 0 | 1 | 1 | 1 |
